# Building a High-Potential Silver–Sulfur Redox Reaction Based on the Hard–Soft Acid–Base Theory

**DOI:** 10.1021/acs.energyfuels.4c00817

**Published:** 2024-05-31

**Authors:** Swati Katiyar, Wentao Hou, Jeileen Luciano Rodriguez, Jose Fernando Florez Gomez, Angelica Del Valle-Perez, Shen Qiu, Songyang Chang, Liz M. Díaz-Vázquez, Lisandro Cunci, Xianyong Wu

**Affiliations:** † Department of Chemistry, University of Puerto Rico-Rio Piedras Campus, San Juan, Puerto Rico 00925-2537, United States; ‡ Department of Physics, University of Puerto Rico-Rio Piedras Campus, San Juan, Puerto Rico 00925-2537, United States

## Abstract

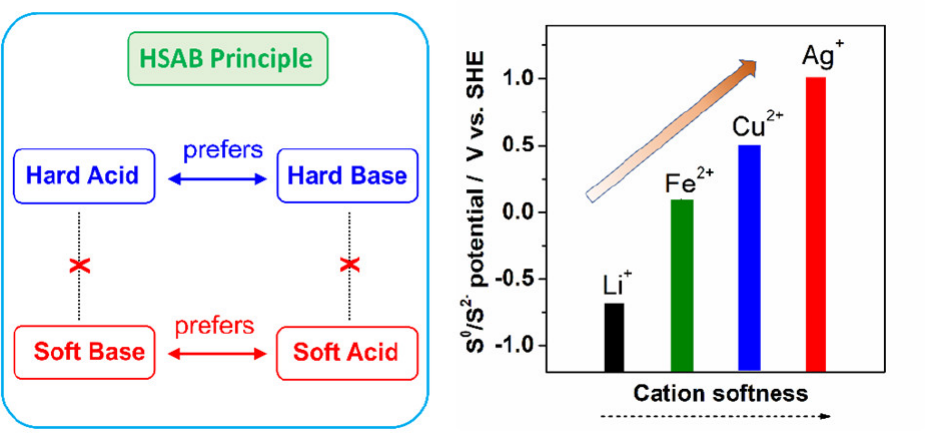

Sulfur holds immense promise for battery applications owing to its abundant availability, low cost, and high capacity. Currently, sulfur is commonly combined with alkali or alkaline earth metals in metal–sulfur batteries. However, these batteries universally face challenges in cycling stability due to the inevitable issue of polysulfide dissolution and shuttling. Additionally, the inferior stability of metal sulfide discharge compounds results in low S^0^/S^2–^ redox potentials (<−0.41 V vs SHE). Herein, we leverage the principle of the hard–soft acid–base theory to introduce a novel silver–sulfur (Ag–S) battery system, which operates on the reaction between the soft acid of Ag^+^ and the soft base of S^2–^. Due to their high reaction affinity, the discharge compound of silver sulfide (Ag_2_S) is intrinsically insoluble and fundamentally stable. This not only resolves the polysulfide dissolution issue but also leads to a predominantly high S^0^/S^2–^ redox potential (+1.0 V vs. SHE). We thus exploit the Ag–S reaction for a primary zinc battery application, which exhibits a high capacity of ∼620 mAh g^–1^ and a high voltage of ∼1.45 V. This work offers valuable insights into the application of classic chemistry theories in the development of innovative energy storage devices.

## Introduction

Rechargeable batteries play an essential role in our modern society. Whether dating back to the historic Daniel battery of 1836 or examining the cutting-edge lithium-ion batteries of today, all batteries rely on redox reactions as the working mechanism for energy storage.^1,2^ This scenario results from the fact that redox reactions are the sole category of chemical reactions capable of electron transfer, which is a fundamental process in electrochemistry and battery operation.^3^ Thus, from a chemistry perspective, it is of critical importance to design innovative redox reactions to build high-performance battery systems.

A battery redox reaction, also referred to as an electrode reaction, involves a host electrode material and an ionic charge carrier.^4^ In general, the electrode material provides the redox center and thus determines the electron transfer number, whereas charge carriers participate in redox reactions to maintain charge neutrality. Among various electrode materials, sulfur represents one of the most compelling candidates, due to its high abundance, low cost, and nontoxicity.^5^ Moreover, the two-electron S^0^/S^2–^ redox reaction leads to its ultrahigh capacity of 1675 mAh g^–1^, surpassing most cathode materials by an order of magnitude.^6^ Consequently, sulfur-based batteries have garnered tremendous attention from researchers worldwide over the past several decades.^7,8^

Presently, sulfur is commonly paired with alkali and alkaline metals to build metal–sulfur (M-S) batteries, including lithium,^7,8^ sodium,^9^ potassium,^10^ magnesium,^11^ and calcium^12^ metal anodes. The objective is to harness the ultralow potentials of these metal anodes, ranging from −2.4 to −3.0 V vs. SHE.^13^ However, the overall M-S cell voltages are moderate, falling between 1.2 and 2.3 V.^7−12^ This indicates a deficiency in the sulfur/sulfide (S^0^/S^2–^) redox potential at the cathode side. Furthermore, due to the solubility of intermediate discharge compounds, these M-S batteries universally face the challenge of polysulfide dissolution and shuttling,^14,15^ which leads to active material loss, rapid capacity fading, and diminished Coulombic efficiency. Although various strategies have been proposed to tackle this challenge, the practical implementations of M-S batteries remain problematic.^16^

**Figure 1:**
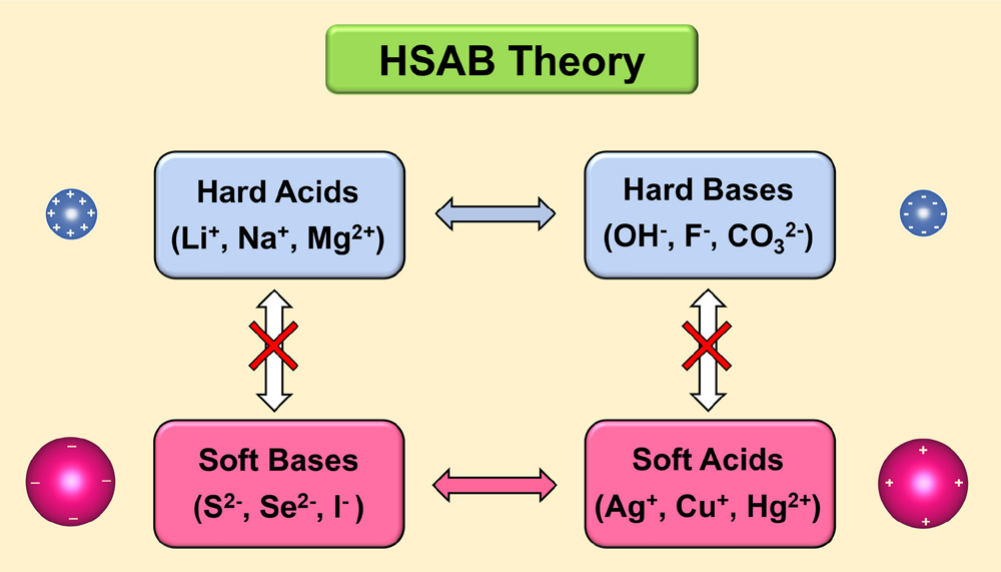
Scheme of the hard–soft acid–base (HSAB) theory.

As battery chemists, we aim to leverage the understanding of classic chemistry theories to devise innovative metal–sulfur batteries, which feature unique properties that were unattainable in previous sulfur systems. We underline that hard–soft acid–base (HSAB) principle is a prominent theory (Figure 1) in textbooks,^17,18^ which qualitatively describes the reaction affinity, mechanism, and stability of acid–base reactions. It categorizes atoms or ions into two major types: soft and hard. A soft acid/base typically exhibits a large size, a low oxidization state, and good polarizability, whereas a hard acid/base tends to have a small size, a high oxidization state, and poor polarizability. Additionally, this theory posits that a soft acid prefers to bind with a soft base, while a hard acid prefers a hard base, both leading to the formation of stable product compounds.^17,18^

This principle provides valuable implications in the context of M-S batteries. Notably, when sulfur is reduced to the S^2–^ anion, it pertains to a soft base, which disfavors hard acids like Li^+^, Na^+^, K^+^, Mg^2+^, and Ca^2+^.^18^ This aversion results in the formation of less stable metal sulfide compounds with higher solubility. For example, Li_2_S and Na_2_S are soluble in water, prone to hydrolysis reactions, and release hydrogen sulfide in air. The less favorable binding between these cations and S^2–^ anions sheds lights on the challenges in building M-S batteries. By contrast, soft acids, such as Cu^+^, Ag^+^, and Hg^2+^, exhibit a high affinity for binding with the S^2–^ soft base, forming stable compounds.^17^ For instance, copper(I) sulfide (Cu_2_S) constitutes a major component in the chalcocite mineral,^19^ existing on Earth for millions of years. Similarly, silver sulfide (Ag_2_S) naturally forms on the surface of silverware, even in the presence of trace amounts of sulfur species in the air.^20^ When a mercury-based thermometer is broken, it is a common sense to use sulfur to treat the mercury waste splits.^21^

Recently, researchers have started to explore transition-metal–sulfur (TM-S) batteries,^22−38^ which employ borderline acids (Fe^2+^, Zn^2+^, Cu^2+^, and Pb^2+^) as ionic charge carriers. Compared with conventional M-S batteries using hard acid ions, these TM-S batteries exhibit intriguing properties, such as higher reaction potentials (0–0.5 V vs. SHE), elimination of soluble polysulfides, and stable cycling performance.^22−38^ In 2019, Ji et al. investigated TM-S batteries, where various cations were compared, including Fe^2+^, Ni^2+^, Co^2+^, Mn^2+^, and Pb^2+^. Among them, the Fe–S battery delivers the optimal performance, with a high capacity of ∼1050 mAh g^–1^, a S^0^/S^2–^ potential of ∼0 V vs. SHE, and robust cycling for 150 cycles.^22^ Later, Ji et al. discovered that the Cu–S battery experienced a unique four-electron transfer reaction, leading to an ultrahigh capacity of ∼2700 mAh g^–1^ and a high S^0^/S^2–^ potential of ∼0.5 V vs. SHE.^23^ In 2021, Shu et al. comprehensively analyzed theoretical reaction thermodynamics of various aqueous metal–sulfur batteries, wherein 316 battery systems and 63 Pourbaix-diagrams were discussed.^30^ Recently, Chao et al. provided an insightful review on aqueous sulfur battery performances and working mechanisms.^38^ Nevertheless, to our knowledge, there has been no experimental report of coupling the S^2–^ soft base and soft acids (Cu^+^, Ag^+^, and Hg^2+^) yet. It remains uncertain whether these innovative redox reactions are viable for battery applications.

Among these soft acids, Hg^2+^ ions are highly toxic, whereas Cu^+^ ions are unstable in aqueous electrolytes, because they undergo a spontaneous disproportionation reaction (2Cu^+^ → Cu^2+^ + Cu). In comparison, Ag^+^ salts have low toxicity and are readily available. Therefore, in this work, we conceived an insoluble and high-potential silver–sulfur (Ag–S) battery based on the HSAB theory. Thanks to the favorable binding between the Ag^+^ soft acid and S^2–^ soft base, the resultant Ag_2_S discharge compound is highly insoluble in water, boasting an ultralow solubility product constant (K_sp_) of 8 × 10^–51^.^39^ This fundamentally resolves the polysulfide dissolution issue. Furthermore, the low Gibbs free energy state of Ag_2_S imparts an ultrahigh S^0^/S^2–^ (S/Ag_2_S) redox potential of +1.0 V vs SHE, exceeding the conventional S/Li_2_S redox couple by 1.7 V. To harness this elevated potential, we integrate the Ag–S redox with a zinc metal in a hybrid battery, which delivers a high capacity of ∼620 mAh g^–1^ (based on sulfur) and a high voltage of ∼1.45 V.

## Results and Discussions

We employed the melt diffusion method^40^ to prepare the sulfur/Ketjen black (S/KB) composite with a designated sulfur loading of 60 wt %. Figure S1 presents basic characterizations of the S/KB sample, including the thermogravimetric analysis (TGA) and X-ray diffraction (XRD) pattern. Electrochemical characterizations of the Ag–S redox reaction were conducted in two-electrode Swagelok cells, with titanium rods serving as current collectors. The working electrode, reference/counter electrode, and the electrolyte is the S/KB, a silver powder paste, and 1 mol L^–1^ AgNO_3_ aqueous solution, respectively. Note that conventional coin cells are not suitable for testing purposes, because stainless steel cell cases can be corroded by the oxidative Ag^+^ electrolyte.

**Figure 2:**
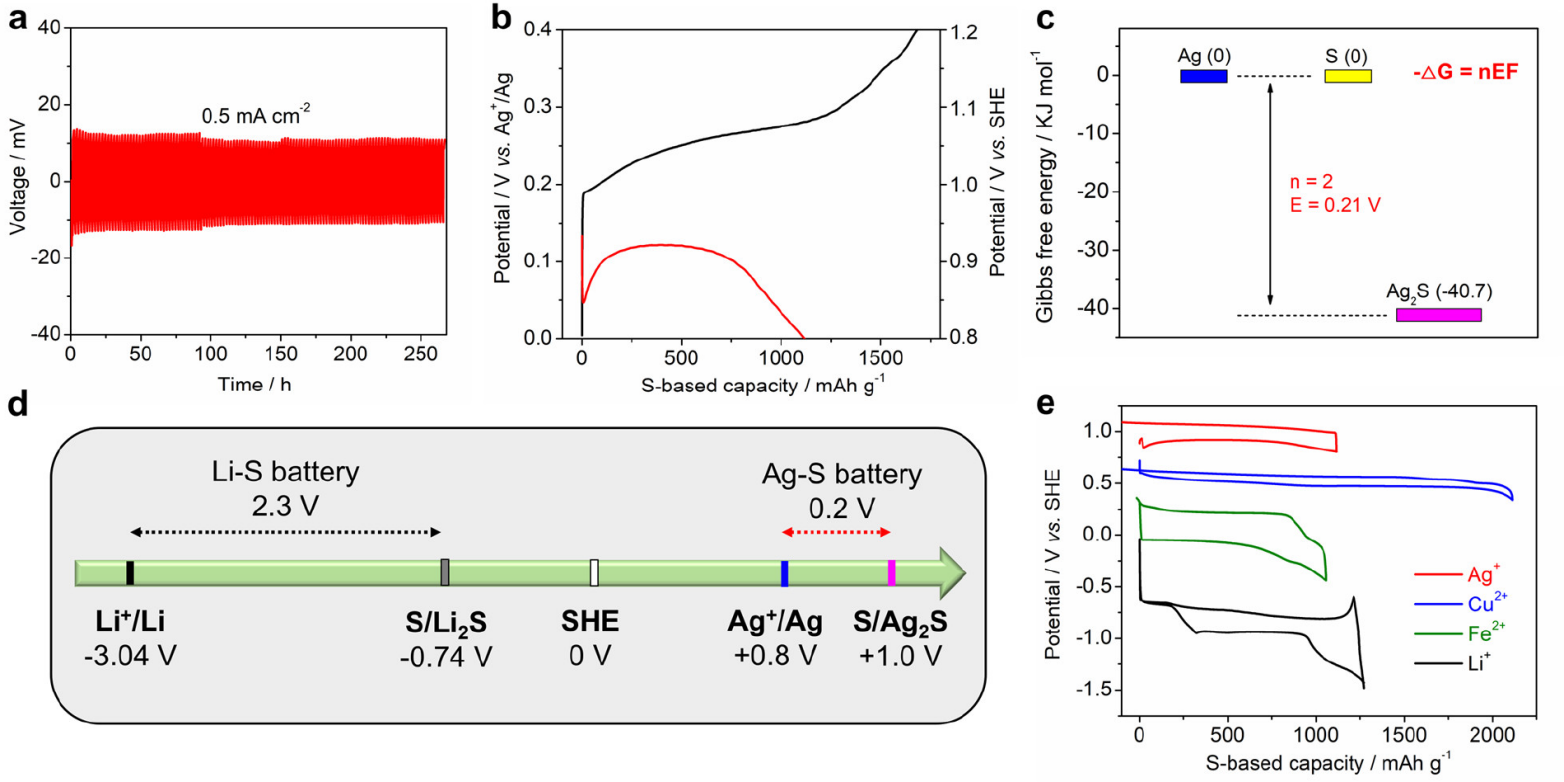
(a) The GCD curves of the symmetrical Ag||Ag battery at 0.5 mA cm^–2^, wherein the plating/stripping capacity is 0.5 mAh cm^–2^ each cycle. (b) The GCD curves of the Ag–S battery at 20 mA g^–1^. (c) The Gibbs free energy states of silver, sulfur, and silver sulfide. (d) The potentials of different redox couples in reference to SHE. (e) The electrochemical performance of the sulfur electrode in storing Ag^+^, Cu^2+^, Fe^2+^, and Li^+^ cations.

Figure 2a displays the Galvanostatic charge/discharge (GCD) curves of symmetrical Ag||Ag batteries. The Ag electrode exhibits minimal polarization of ∼15 mV and sustains long-term cycling for 260 h, without noticeable voltage fluctuations, polarization increments, or cell short-circuits (Figure S2). This indicates that Ag metal is a reliable reference/counter electrode in half-cell studies. Figure 2b shows GCD curves of the Ag–S battery at 20 mA g^–1^. The open-circuit voltage (OCV) is ∼0.14 V. Upon discharge, the S/KB electrode undergoes a sudden potential drop to 0.05 V in the beginning, but subsequently, it stabilizes at 0.12 V and maintains relatively stable in the following discharge process. Given the consistent potential of the Ag electrode, the initial potential drop likely originates from the sulfur electrode, which has low electronic conductivity and contributes to the IR drop. As Ag^+^ ions begin to insert into the sulfur, the resultant Ag_2_S enhances the electronic conductivity of the electrode, leading to an elevation in the reaction potential. It is important to note that the electronic conductivity of Ag_2_S significantly exceeds that of pure sulfur.^41,42^ Indeed, we find that the charge-transfer resistance of the Ag–S battery gets decreased when some Ag^+^ cations are inserted to the sulfur cathode (Figure S3).

The first cycle discharge/charge capacity is 1164/1684 mAh g^–1^, respectively, leading to a moderate Coulombic efficiency (CE) of 69.1%. The AgNO_3_ electrolyte has a neutral pH value, suggesting that the theoretical potential for oxygen evolution reaction is 1.23–0.059*7 = 0.817 V vs. SHE. This potential is lower than the charge cutoff potential in the Ag–S battery (0.4 V vs. Ag^+^/Ag, corresponding to +1.2 V vs. SHE, Figure 2b), leading to certain OER side reactions and a moderate Coulombic efficiency. The charge/discharge potential at the midpoint capacity is approximately 0.28/0.12 V, which gives rise to an average reaction potential of around 0.20 V vs. Ag^+^/Ag. This result agrees well with the thermodynamic data presented in Figure 2c, where the Gibbs free energy of Ag, S, and Ag_2_S is 0, 0, and −40.7 kJ mol^–1^, respectively.^43^ As such, the total Gibbs free energy change (Δ*G*) for the reaction of 2Ag + S = Ag_2_S is −40.7 kJ mol^–1^. Utilizing the fundamental equation −Δ*G* = nEF,^39^ we calculate the reaction potential E as 40.7/(2*96.485) = 0.21 V, which is in close agreement to the experimental data (0.20 V). Here, n and F denote the electron transfer number and Faraday constant, which is 2 and 96.485 KJ V^–1^, respectively.

The Ag–S system exhibits a modest cell voltage of +0.2 V, which seems inferior to the widely studied Li–S batteries. However, when we differentiate between “cell voltage” and “electrode potential”, the distinctive nature of the Ag–S redox system becomes apparent. It is known that the cell voltage is a result of electrode potential differences between cathode and anode, and the electrode potential needs to be specified with a reference electrode. The Li–S battery shows a high cell voltage of ∼2.3 V, which primarily stems from the remarkably low potential of the Li metal anode (Li^+^/Li, −3.04 V vs. SHE, Figure 2d).^13^ Consequently, the S/Li_2_S couple exhibits a low redox potential of −0.74 V vs. SHE, which is lower than the standard electrode potential of S/S^2–^ (−0.41 V vs SHE).^13^ In contrast, the Ag^+^/Ag electrode is +0.8 V vs SHE,^13^ which renders a notably high S/Ag_2_S redox potential of +1.0 V vs. SHE (Figure 2d).

For a more comprehensive understanding, we also compared GCD curves of the sulfur electrode when storing other cations. Interestingly, we observe an increase in the S/S^2–^ redox potential in the order of Ag^+^ > Cu^2+^ > Fe^2+^ > Li^+^ (Figure 2e). This trend finds a compelling explanation within the framework of the HSAB theory. When sulfur is reduced to sulfide, it falls into the category of a soft base, which prefers to bind with soft acids, such as Ag^+^. The resulting Ag_2_S compound is thermodynamically stable with a low Gibbs free energy, leading to an increase in the reaction potential as return. The S^2–^ anion can also bind with borderline acids, such as Cu^2+^ or Fe^2+^, but the resulting compounds are relatively less stable, resulting in moderate redox potentials. Conversely, if sulfide is bound with hard acids, like Li^+^, Na^+^, and K^+^, the resulting sulfide compounds are the least stable, which exhibit low redox potential. Overall, when Ag^+^ ions are used as charge carriers, the S/S^2–^ redox potential surpasses the Li–S system by 1.7 V, representing a significant improvement for the sulfur redox reaction. It is noted that the Cu–S battery delivers a high capacity of ∼2000 mAh g^–1^, which is due to the unique 4-electron transfer reaction as reported previously.^23^

**Figure 3:**
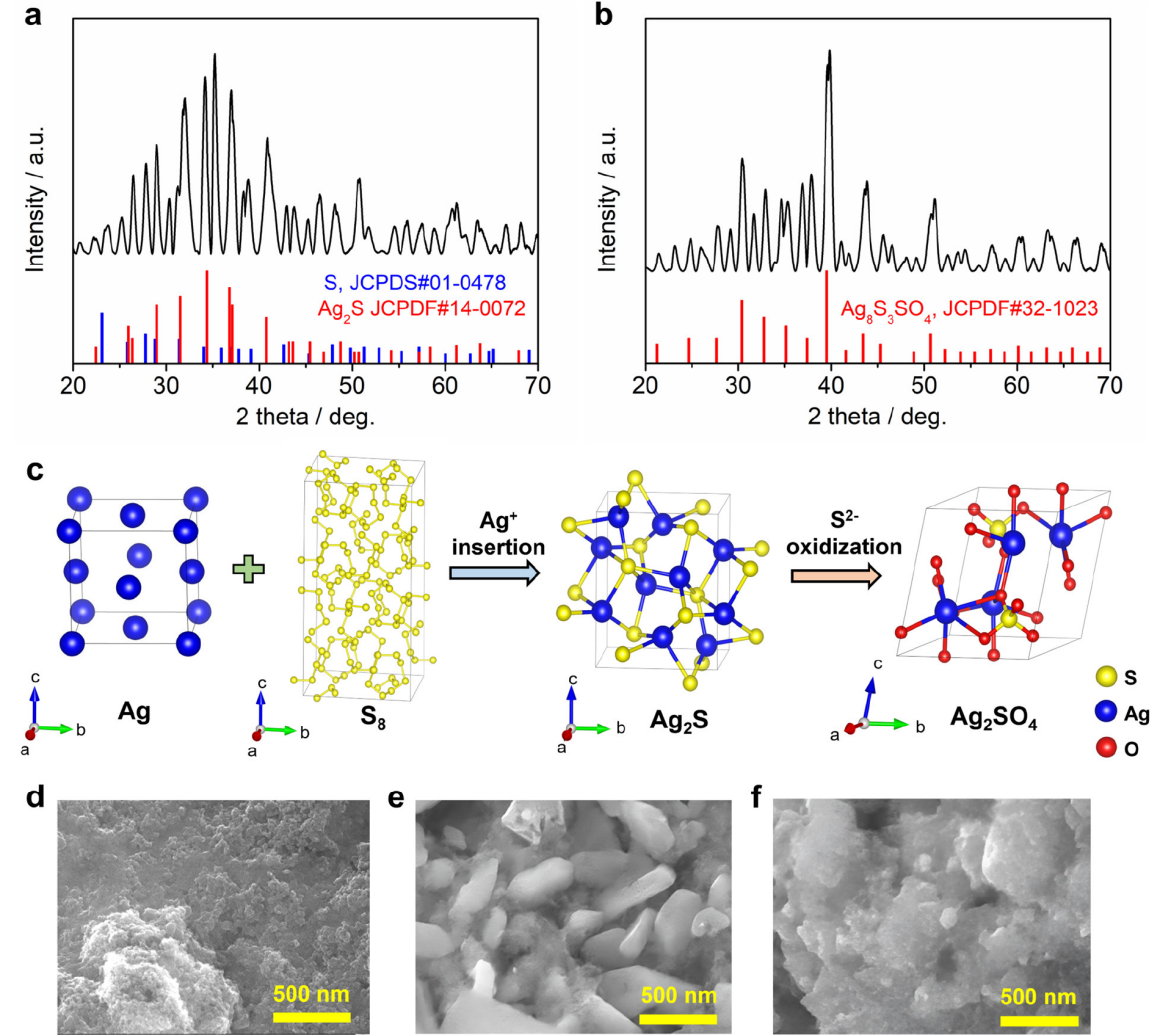
*Ex situ* XRD patterns at (a) charged state and (b) discharged state. (c) Schematics of the unit cells of Ag, S_8_, Ag_2_S, and Ag_2_SO_4_, showing the conversion pathways of the electrode. SEM image of electrodes at (d) pristine S/KB, (e) discharge state, and (f) charged state.

Despite its exceptionally high redox potential, this Ag–S reaction is irreversible, which supports only one cycle of charge/discharge. Subsequently, the reaction capacity diminishes to ∼15 mAh g^–1^ (Figure S4). To understand the reaction mechanism, we carried out *ex situ* XRD experiments to investigate the structure evolution. We physically mixed the crystalline sulfur with Ketjen black carbon and made a self-standing film using a polytetrafluoroethylene (PTFE) binder. Figure 3a presents the XRD pattern of the fully discharge electrode, which can be indexed to a composite material of sulfur (JCPDS#01–0478) and Ag_2_S (JCPDS#14–0072). This result substainitiates our hypothesis of the Ag_2_S formation. It is noteworthy that there is no other form of silver sulfide in the crystal structure database, and Ag_2_S is the only form of silver sulfide, which is an insoluble precipitate with ultralow K_sp_ of 8 × 10^–51^. In the 1 M Ag^+^ electrolyte, the sulfide concentration is water is reduced to 8 × 10^–51^ M due to the common ion effect (see the calculation in the experimental section). Therefore, by using Ag^+^ as the charge carrier, we can fundamentally eliminate the infamous polysulfide dissolution/shuttling issue.

The fully charged electrode primarily comprises a silver sulfide sulfate material (Ag_8_S_3_SO_4_, JCPDS # 32–1023), as shown in Figure 3b. This means that upon charging, S^2–^ anions in Ag_2_S cannot reversibly oxidized back to sulfur. Instead, 1/4 of S^2–^ anions experience overoxidization and further react with water to form [SO_4_]^2–^ anions. A similar phenomenon has been reported in the Zn–S batteries,^24,25^ where ZnS is oxidized to ZnSO_4_ at a cutoff potential of 1.5 V vs. Zn^2+^/Zn. Sulfate materials are generally redox-inert in battery studies, which eventually terminate the Ag–S reaction.

To provide a visual representation of the reaction mechanism, we have illustrated the structural evolution of S, Ag_2_S, and Ag_2_SO_4_ in Figure 3c. Since the crystallographic information file (CIF) of Ag_8_S_3_SO_4_ is unavailable, we used the Ag_2_SO_4_ CIF as an alternative, which represents 1/4 of the Ag_8_S_3_SO_4_ phase: [Ag_2_S]_3_·[Ag_2_SO_4_]. Interestingly, the discharge process corresponds to cation (Ag^+^) insertion, whereas the charge process involves anion (O^2–^) insertion.

Scanning electron microscopy (SEM) further affirms the irrversible morphology change (Figure 3d-f). In the pristine electrode, numerous regularly shaped nanosized materials are evident, which are attributed to the S/KB composites. Following discharge, pellet-like materials emerge in the sample (Figure 3e and Figure S5). The energy dispersive spectra (EDS) and mapping result reveals that the Ag/S molar ratio is approximately 2:1 (Figure S6), which suggests the Ag_2_S formation. Upon the charge process, these pellets vanish, and some quasi-cubic materials start showing up (Figure 3f and Figures S7–S8). Overall, the morphology at the charged state markedly differs from that of the pristine S/KB sample, which provides additional evidence of the reaction irreversibility. EIS tests revealed that the charge-transfer resistance was progressively increased at higher states of charge (Figure S9), which corroborated XRD and SEM results.

To mitigate the sulfide oxidization issue and demonstrate a reverisble Ag–S redox reaction, we increased the AgNO_3_ concentration from 1 m (mol kg^–1^) to 2, 5, and 10 m, hoping concentrated or “Water-in-Salt” (WiS) electrolytes could suppress the water reaction activity and thus improve the reaction reversibility.^44−46^ However, the Ag–S battery shows low discharge capacities of ∼850, ∼ 700, and ∼180 mAh g^–1^ in 2, 5, and 10 m AgNO_3_ electrolyte, respectively, and the overall reactions are still not reversible (Figure S10). It is likely that these electrolytes still contain a significant amount of free water molecules, which can react with sulfide to form sulfate ions. Notably, in the 10 m AgNO_3_ electrolyte, the molar ratio between Ag^+^ ions and H_2_O molecules is 10:55, which may not be sufficient to completely bind all the water molecules. Consequently, we conclude that the Ag–S reaction exhibits a very high redox potential, but unfortuantely, it incurs side reactions with water, eventually leading to the reaction irreversibility. Future studies will be focused on the development of novel WiS electrolytes with a higher molality, which exhibits even less water molecules. Alternatively, nonaqueous electrolytes could be explored, such as silver perclorate or hexafluorophosphate in carbonate solvents, which contain no water molecules. These approaches may mitigate the sulfide-water side reaction and thus benefit the reaction reversibility. The discovery here also implies that there is a balance between the reaction potential and reversibility for the sulfur electrode, where the soft acid (such as Ag^+^) features a high redox potential but poor reversibility, whereas the borderline acid (Cu^2+^ or Fe^2+^) shows a moderate potential but excellent reversibility.

The rate performance of the Ag–S battery is provided in Figure S11, where a similarly high capacity of ∼1131 mAh g^–1^ is obtained at 50 mA g^–1^. However, at higher current rates (75 and 100 mA g^–1^), the potential drop issue occurs again, leading to a minimal discharge capacity. This suggests that Ag–S battery has moderate reaction kinetics, which is more suitable for low-power and low-current primary battery applications, as compared in Table S1.

To exploit the high Ag–S redox potential, we work to demonstrate a primary battery with a high capacity and a good voltage. It is worth mentioning that in the context of primary batteries, the silver oxide battery (Zn||Ag_2_O) finds wide applications in miniature devices,^47,48^ such as watches, calculators, and hearing aids. This battery features a good voltage of ∼1.5 V, a reasonable capacity of ∼230 mAh g^–1^, and high volumetric energy density due to the high density of silver-based compounds.^48^ Motivated by these properties, we assembled a hybrid Zn||Ag–S battery with the aid of an anion-exchange membrane, which can take advantage of the high capacity and high potential of the Ag–S redox reaction.

**Figure 4:**
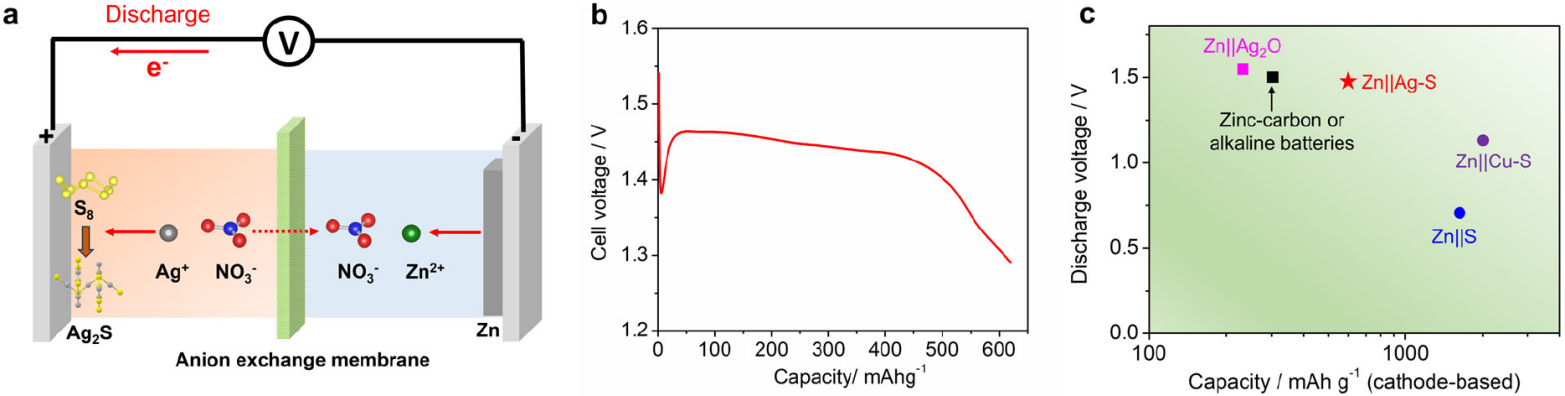
Hybrid Zn||Ag–S battery. (a) The working mechanism. (b) The discharge curve at 50 mA g^–1^. (c) The capacity–voltage comparison with similar battery systems. Note that the Zn||Cu–S battery utilizes an aqueous CuSO_4_ electrolyte, which contains borderline acids of Cu^2+^ instead of soft acids of Cu^+^ ions in the solution.

Figure 4a shows the working mechanism of the hybrid Zn||Ag–S battery, where the cathode reaction works on the Ag^+^ insertion in the sulfur structure, whereas the anode reaction operates on the Zn metal stripping. To balance the charge, NO_3_^–^ anions serve as ionic charge carriers and migrate from the AgNO_3_ to Zn(NO_3_)_2_ electrolyte. Figure 4b shows the discharge curve of the hybrid battery at 50 mA g^–1^, where a high capacity of ∼620 mAh g^–1^ (sulfur-based) and a good voltage of ∼1.45 V is attainable. There is an initial potential drop in this hybrid battery, and it primarily results from the potential drop of the sulfur cathode, which has been observed in the Ag–S half cell studies (Figure 2b). Based on the sulfur active mass, the specific energy can reach ∼900 Wh kg^–1^. For a better understanding, we also compared the Zn||Ag–S battery performance with primary zinc batteries (zinc–carbon, alkaline zinc, and zinc–silver-oxide) as well as relevant S-based zinc batteries. As shown in Figure 4c and Table S2, our battery exhibits a much higher cell voltage (1.45 V) than Zn–S batteries (0.7 V)^23^ and Zn||Cu–S batteries (1.15 V, Cu^2+^ ions in the electrolyte),^24^ which is also on par with commercial primary zinc batteries (1.5–1.55 V). Besides, our cathode delivers a higher specific capacity of 620 mAh g^–1^ than commercial MnO_2_ cathodes (230–308 mAh g^–1^) in zinc–carbon and alkaline zinc batteries, showing alternative potential for primary battery use.

We note that the lower capacity in the hybrid cell may result from two reasons. First, the self-standing S/KB electrode in the hybrid cell exhibits a much higher mass loading of ∼15 mg cm^–2^. Second, the relatively thick anion-exchange membrane will contribute to a higher charge-transfer resistance than that in Ag–S half-cells. This hybrid battery aims to provide an alternative approach to incorporate novel redox reactions for battery applications, instead of fabricating practical batteries. It is also important to note that in practical batteries, numerous parameters require optimization, and all material masses should be considered to calculate the energy density of the full cell.^49−52^

Besides the primary battery application, this Ag–S reaction also provides a novel approach to synthesize Ag_2_S@C (or Ag_8_S_3_SO_4_@C) nanocomposites in a simple and mild aqueous medium, and these materials may find alternative applications for energy storage or in other areas. As shown in Figure S12, we retrieved the electrode after discharging the Ag–S battery, which exhibited a characteristic GCD curve of Ag_2_S in Li-ion batteries, thus confirming the feasibility of this approach.

## Conclusion

In this work, we leveraged the understanding of the HSAB theory and designed a unique Ag–S redox reaction, which features a predominantly high S/S^2–^ redox potential (+1.0 V vs. SHE) and a solid–solid conversion reaction mechanism that avoids the polysulfide dissolution issue. However, such a high potential triggers the side reaction between sulfide and water, where the Ag_2_S is overoxidized to a redox-inert Ag_8_S_3_SO_4_ phase, thus terminating the subsequent electrochemical reactions. To exploit the high potential of the Ag–S redox, we implemented an aqueous hybrid Zn||Ag–S primary battery, which realized a high capacity of ∼620 mAh g^–1^ and a promising voltage of ∼1.45 V. Our work exemplifies the power of utilizing fundamental chemistry principles to design innovative redox reactions.

## Supporting Information Available

Experimental details on the material/electrode preparation. Physical and electrochemical characterization (XRD, TGA, SEM, charge/discharge curves, EIS) (PDF)
