# Supplementary material for: Building a High-Potential Silver–Sulfur Redox Reaction Based on the Hard–Soft Acid–Base Theory
Source: Energy Fuels. 2024 May 31;38(12):11233–9. doi: 10.1021/acs.energyfuels.4c00817 (PMC11194820; doi:10.1021/acs.energyfuels.4c00817)
Supplement: Supplementary file 1 — ef4c00817_si_001.pdf [file ef4c00817_si_001.pdf]

Supporting information for

**Building A High-Potential Silver-Sulfur Redox Reaction Based on the Hard-Soft Acid-Base Theory**

Swati Katiyar,[a] Wentao Hou,[a] Jeileen Luciano Rodriguez,[a] Jose Fernando Florez Gomez,[b] Angelica Del Valle-Perez,[a] Shen Qiu,[a] Songyang Chang,[a] Liz M. Díaz-Vázquez,[a] Lisandro Cunci,[a] and Xianyong Wu*[a]

a Department of Chemistry, University of Puerto Rico-Rio Piedras Campus, San Juan, PR 00925-2537, USA

b Department of Physics, University of Puerto Rico-Rio Piedras Campus, San Juan, PR, 00925-2537, USA

Email: xianyong.wu@upr.edu

**Experimental methods**

**1. Material synthesis and electrode preparation**

The sulfur/Ketjen black nanocomposite (S/KB) was prepared by the melt-diffusion method. The sulfur (0.6 g) powders were first ground with Ketjen black (KB, 0.4 g) in a mortar for 30 minutes, and the resultant composite mixture was transferred to a planetary ball milling jar and subjected to ball milling for 5 hours at 300 rpm. We collected the composite and pressed it into a pellet, which was transferred to a sealed autoclave. The melt-diffusion reaction takes place at 155 °C for 6 hours. When the reaction finishes and cools down, the S/KB composite was ground into fine powders for use.

The S/KB electrode comprises the S/KB-60 composite and polyvinylidene fluoride (PVDF) binder in a mass ratio of 9:1. The slurry was made with help of the N-Methyl-2-pyrrolidone solvent, which was further cast on carbon fiber papers (Fuel Cell Store, AvCarb MGL370). The carbon fiber thickness is 0.37 mm, and the diameter is 1 cm in diameter. The S/KB electrodes were dried in an air-forced oven at 45 ℃ for 12 hours, and the electrode mass loading is 1.5-2.0 mg $cm^{-2}$. For *ex-situ* XRD tests, the crystalline sulfur powder was ground with Ketjen carbon in an 8:1 mass ratio, and then the composite was mixed with polytetrafluoroethylene (PTFE) binder in 8:1:1 mass ratio. which was further rolled into a self-standing film. The silver reference and counter electrode is also a self-standing film, which comprises 80 wt.% silver powders, 10 wt.% carbon, and 10 wt.% PTFE binder.

**2. Physical characterization**

X-ray diffraction (XRD) patterns of the S/KB powders were collected on the Rigaku MiniFlex Ⅱ powder diffractometer. The XRD patterns of the self-standing film electrodes were collected on the Rigaku SuperNova equipped with a HyPix3000 X-ray detector and CuKα radiation source (λ = 1.5406 Å). Scanning electron microscopy (SEM) images were recorded at a field emission scanning electron microscope (SEM, JEOL, JSM-6480LV). The thermogravimetric analysis (TGA) of S/KB was carried out on the Mettler-Toledo TGA 2 instrument.

**3. Battery assembly and testing**

The Ag-S and symmetrical silver batteries were assembled in Swagelok cells, which use titanium rods as the current collectors. The electrolyte is an aqueous 1.0 M $AgNO_3$ solution,

and the volume is 100 μL. The hybrid battery was assembled in a breaker cell configuration, where the zinc metal was immersed in the 1 M $Zn(NO_3)_3$ electrolyte, and the S/KB self-standing electrode (pressed onto a titanium mesh) was immersed in the 1 M $AgNO_3$ electrolyte. The supporting electrolyte was 1 M $KNO_3$ electrolyte. To connect the Zn anode and the Ag-S cathode reaction, we used anion-exchange membranes (fumasep®FAB-PK-130, Fullcell Store, thickness 130 μm) to connect these two electrode compartments and prevent the $Zn^{2+}$ and $Ag^+$ electrolytes from mixing with each other. For Li-S batteries, the counter/reference electrode is the lithium metal, and the electrolyte is 1.0 M LiTFSI/DOL-DME with 2.0% $LiNO_3$ additive. For Fe-S batteries, the counter/reference electrode is an iron powder electrode cast on carbon fiber papers, and the electrolyte is 0.5 M $FeSO_4$ aqueous solution. For Cu-S batteries, the counter/reference electrode is a piece of copper foil, and the electrolyte is 0.5 M $CuSO_4$ aqueous solution. To make the $Ag_2S$ battery, we first discharged the sulfur electrode in the $AgNO_3$ electrolyte and then retrieved it with cleaning and drying. The Li-$Ag_2S$ battery was made in a glove box, where the counter/reference electrode is the Li metal, and the cathode is the $Ag_2S$ electrode. The electrolyte is 1.0 M LiTFSI/DOL-DME with 2.0% $LiNO_3$ additive.

For ex-situ SEM and XRD tests, the electrodes have been thoroughly washed by water and ethanol many times. The Galvanostatic charge/discharge tests were performed on the Landt battery tester (CT3002AU) at room temperature.

**4. Calculation of the solubility of sulfides in 1 M $AgNO_3$**

The dissolution equilibrium for $Ag_2S$ can be expressed as: $Ag_2S$ (s) $\rightleftharpoons$ $2Ag^+$ (aq) + $S^{2-}$ (aq). Therefore, the solubility product constant $K_{sp}$ can be expressed as $K_{sp} = [Ag^+]^2 \cdot [S^{2-}]$, where $[Ag^+]$ and $[S^{2-}]$ is the molar concentration of silver and sulfide ions in the solution, respectively. Considering the common ion effect and the electrolyte of 1 M $AgNO_3$, we can use 1 M as the $[Ag^+]$ concentration and ignore the contribution from the $Ag_2S$. Therefore, $K_{sp} = 1^2 \cdot [S^{2-}] = 8\times10^{-51}$. The sulfide concentration is calculated as low as $8\times10^{-51}$ M, which can fundamentally avoid the polysulfide dissolution and shuttling issue.

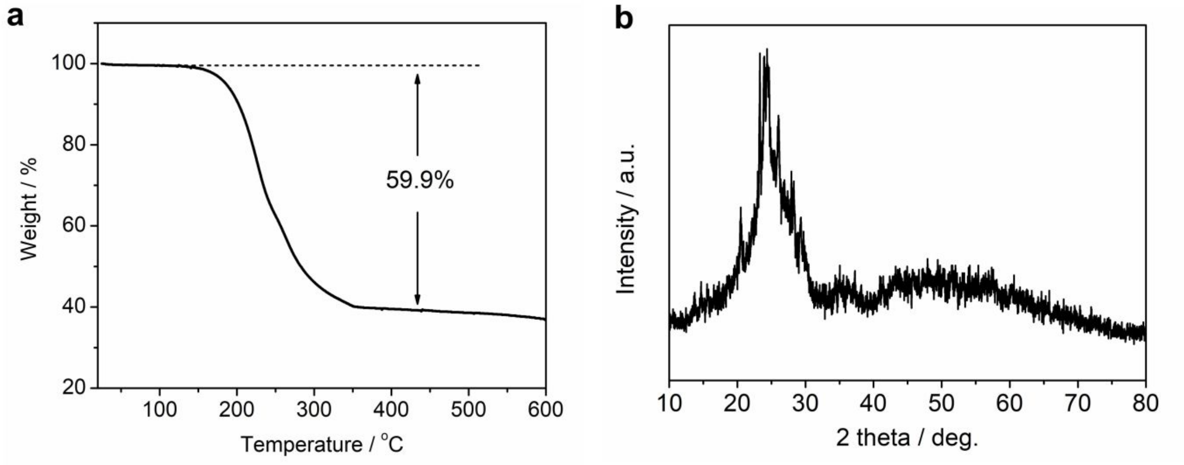


**Figure S1**. Physical characterization of the S/KB material: (a) The TGA curve of the S/KB in the nitrogen atmosphere tested at a ramp rate of 10 ℃/min; (b) The XRD pattern of the S/KB sample.

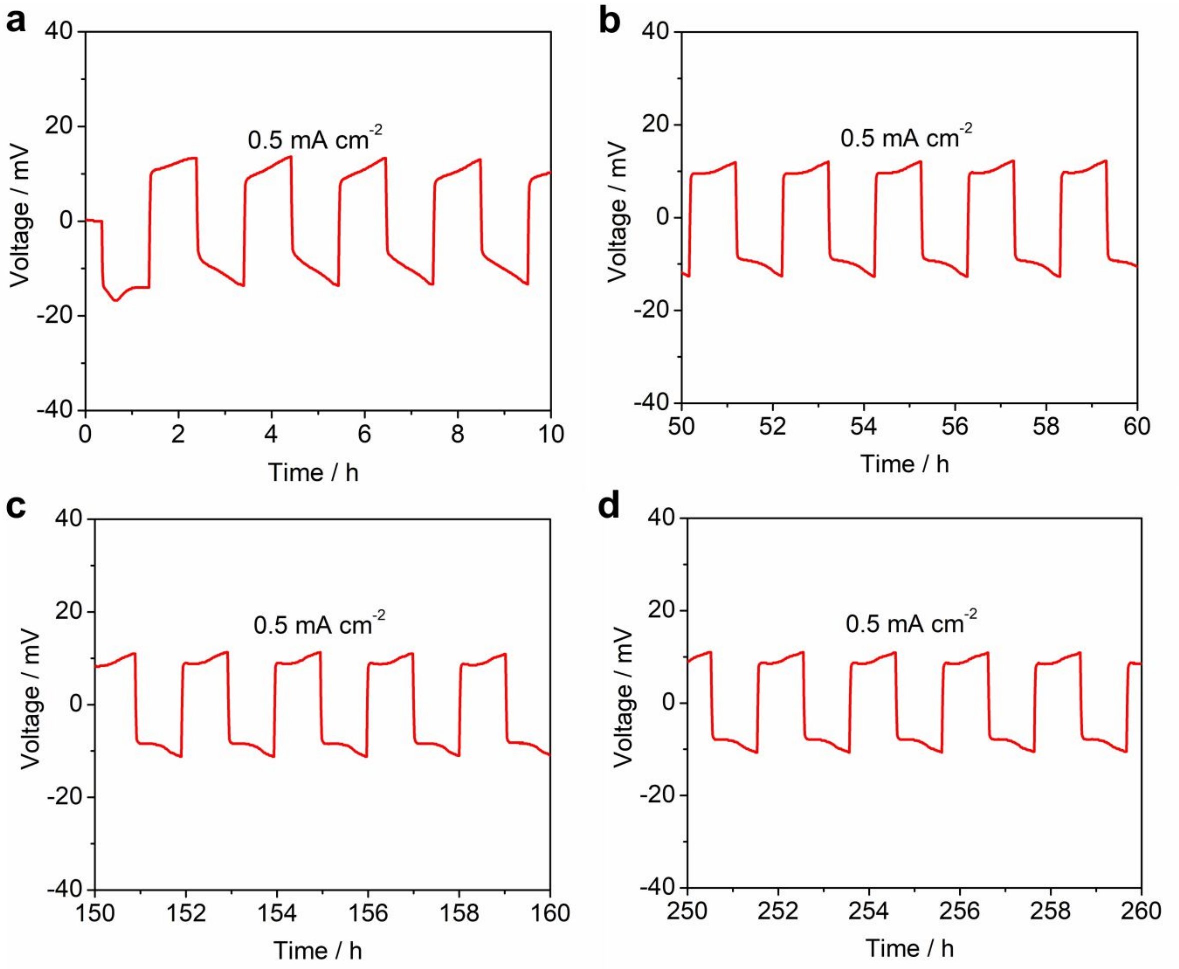


**Figure S2**. The GCD curves of the Ag-Ag symmetrical battery during selected time periods: (a) From the 0 to 10$^{th}$ hour; (b) From the 50$^{th}$ to 60$^{th}$ hour; (c) From the 150$^{th}$ to 160$^{th}$ hour; (d) From the 250$^{th}$ to 260$^{th}$ hour. As shown, there is no severe potential change or cell short circuit, which indicates the reliability and stability of silver as counter/reference electrode.

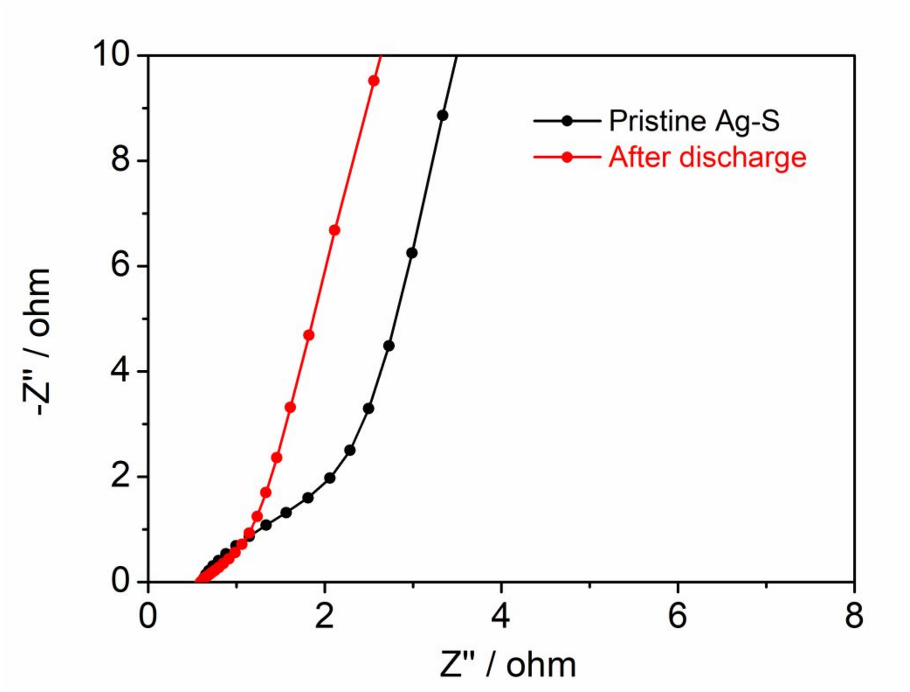


**Figure S3**. The EIS result of the Ag-S battery before and after discharge. The discharge capacity is controlled as 100 mAh $g^{-1}$. As shown, when some $Ag^{+}$ cations insert to the sulfur structure, the charge-transfer resistance gets decreased, which can explain the initial potential drop in the GCD curve.

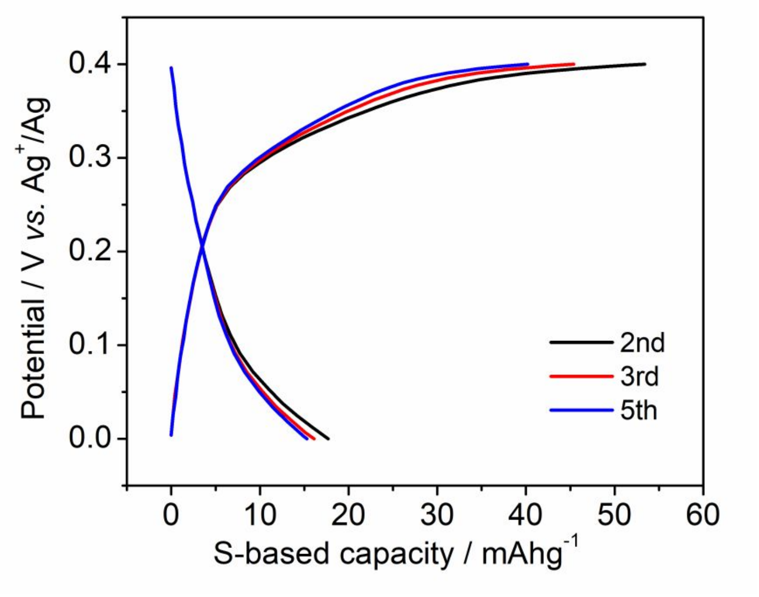


**Figure S4**. The GCD curves of the Ag-S battery in the following 2nd, 3rd, and 5th cycles. As shown, the Coulombic efficiency is constantly low in the subsequent cycles, which is still due to the OER side reaction. There is minimal capacity fading during the discharge cycles, which results from the high stability of the Ketjen black carbon and the ion desorption process.

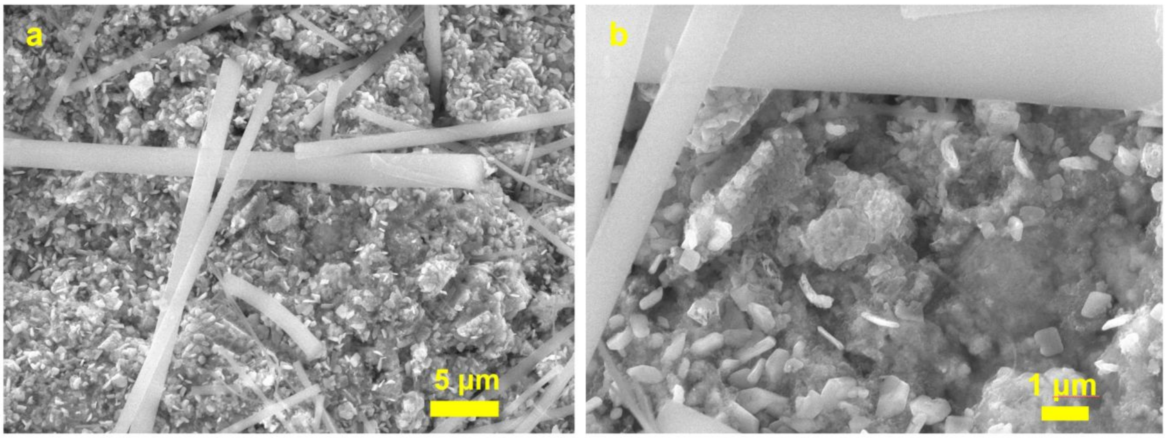


**Figure S5**. The SEM images of the S/KB electrode at the discharged state. (a) The scale bar is 5 μm; (b) The scale bar is 1 μm. The fiber-like material is the glass fiber separator that was stuck onto the electrode surface.

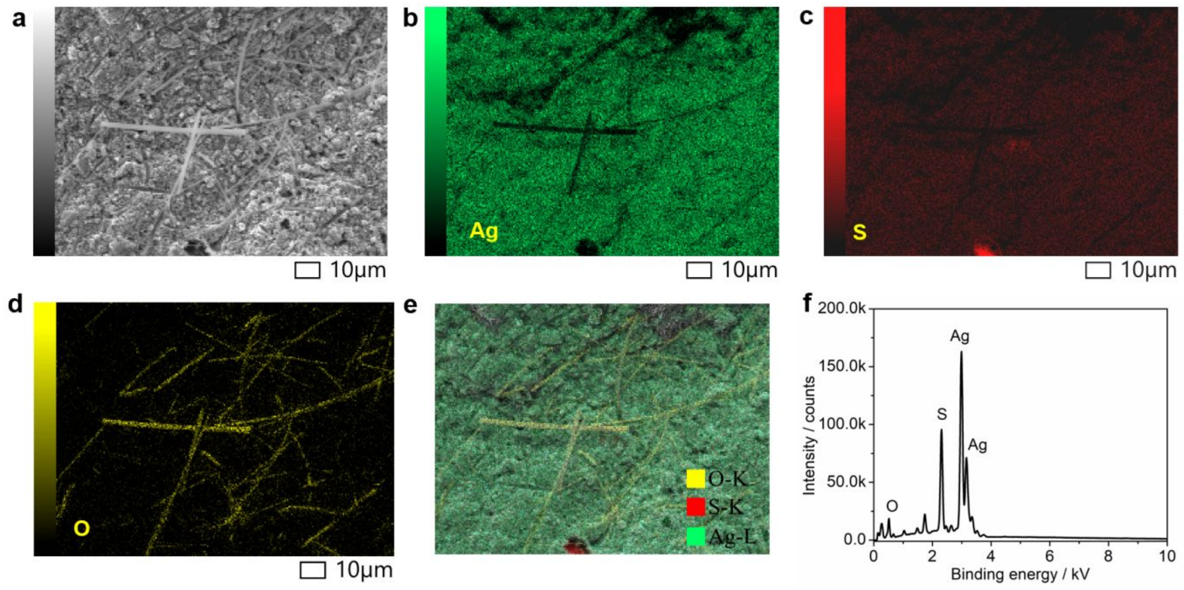


**Figure S6**. The EDS mapping results of the S/KB electrode at the discharged state. (a) The SEM image; (b-d) The Ag, S, and O elemental mapping; (e) The elemental overlapping result; (f) The EDS result. As shown, the silver and sulfur elements are evenly distributed in the discharged electrode, and the Ag/S molar ratio is found to be 1.74:1, close to 2:1 in the $Ag_2S$ formula, which suggests the $Ag_2S$ formation. Of note, the oxygen element is coming from the glass fiber.

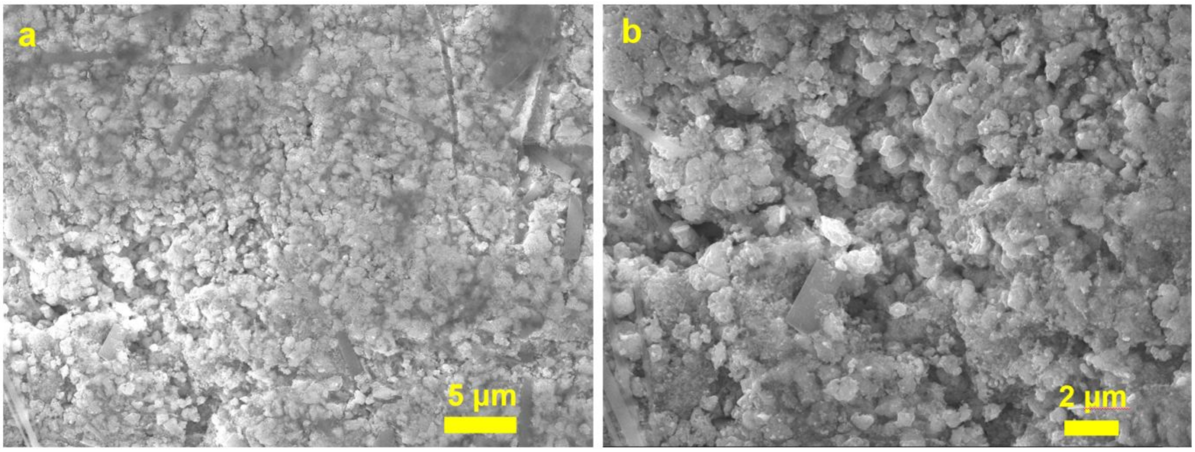


**Figure S7**. The SEM images of the S/KB electrode at the charged state. (a) The scale bar is 5 μm; (b) The scale bar is 2 μm.

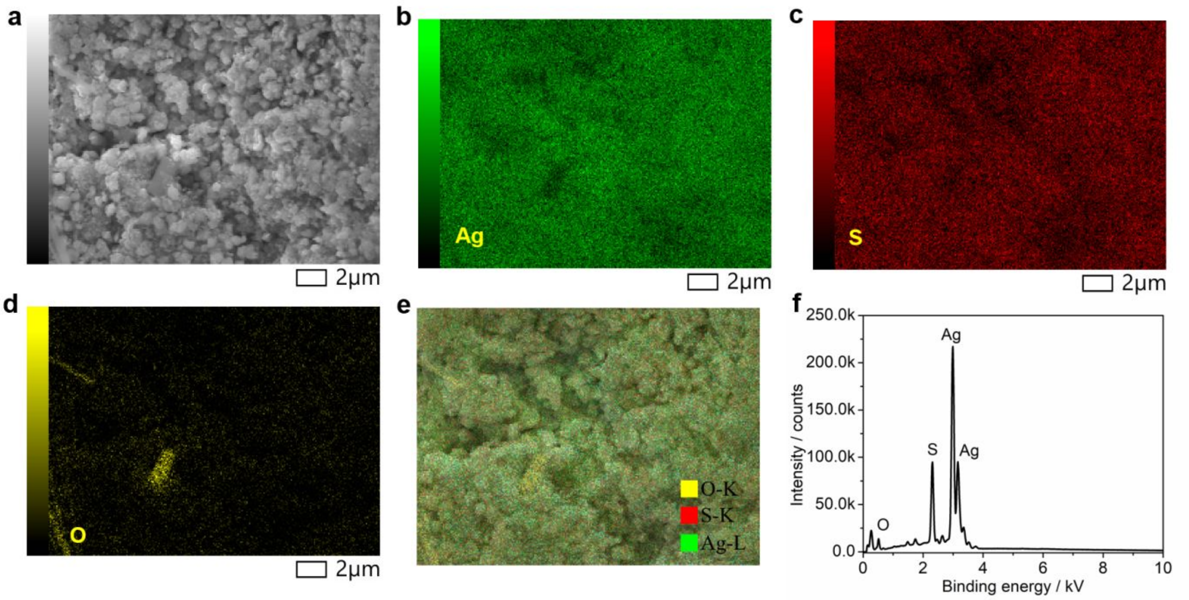


**Figure S8**. The EDS mapping results of the S/KB electrode at the charged state. (a) The SEM image; (b-d) The Ag, S, and O elemental mapping; (e) The elemental overlapping result; (f) The EDS result. As shown, the silver, sulfur, and oxygen elements are evenly distributed in the discharged electrode, and the Ag/S molar ratio is found to be 2.2:1, close to 2:1, which agrees well with the XRD result of the $Ag_8S_3SO_4$ formation.

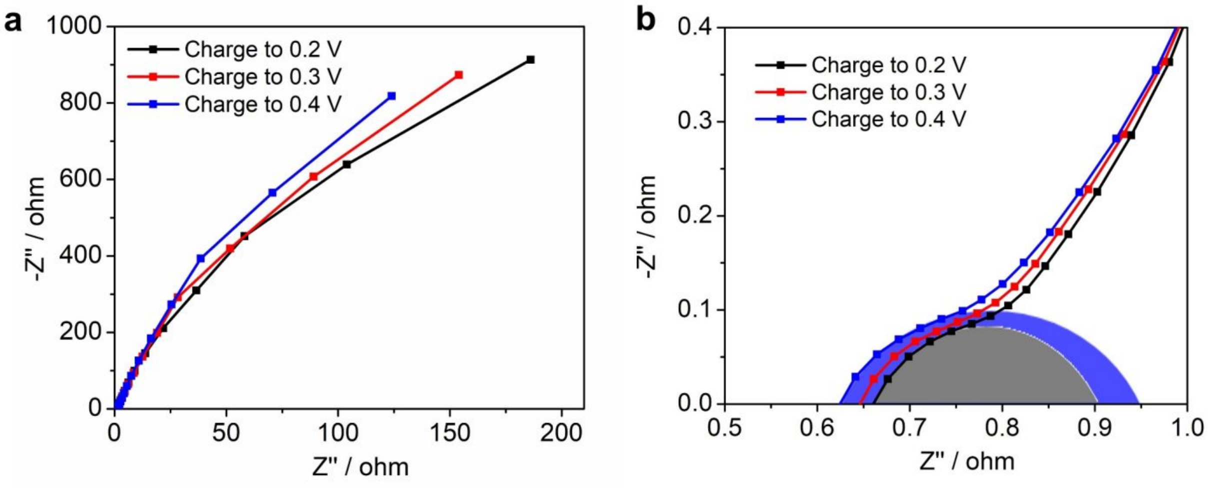


**Figure S9**. The EIS results of the Ag-S battery at different charge voltages. (a) The full EIS spectra; (b) The enlarged area of the EIS result.

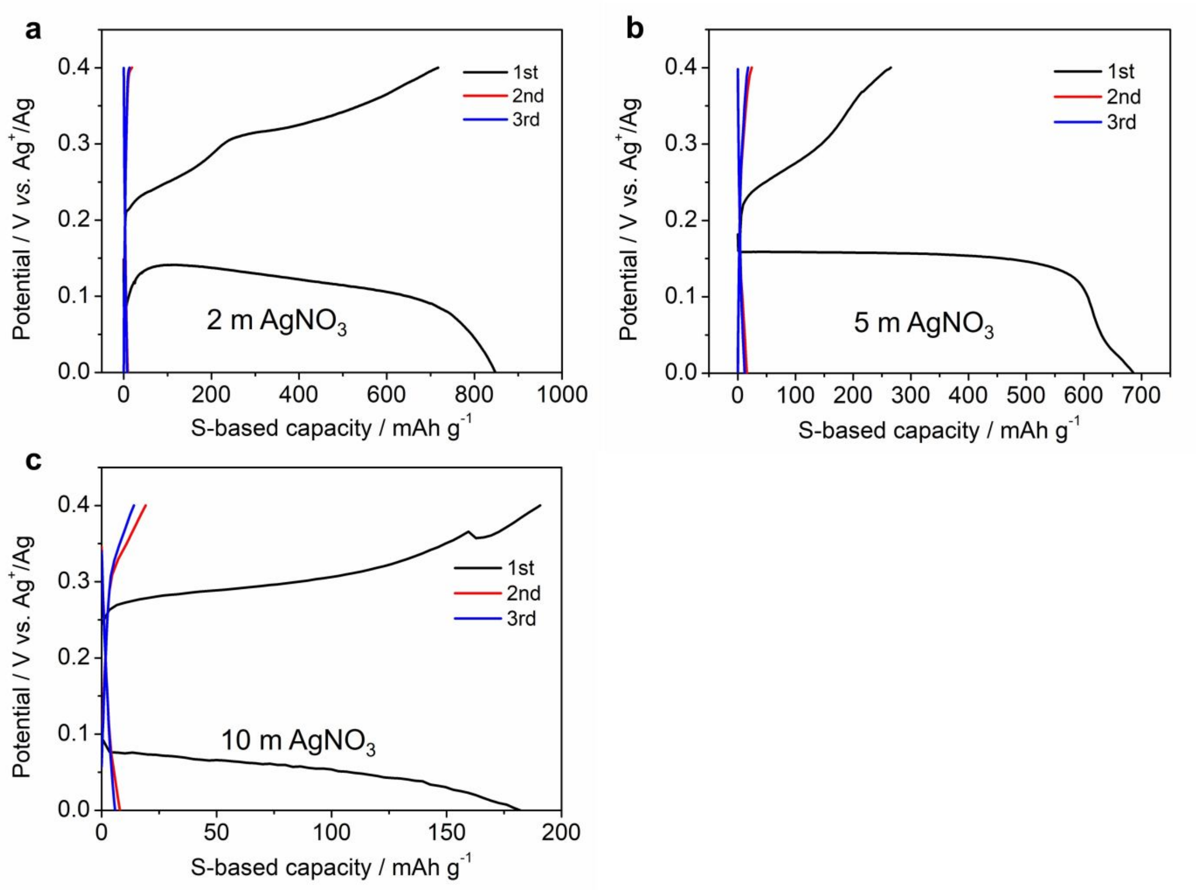


**Figure S10.** The GCD curves of the S/KB electrode in the 2 m, 5 m, and 10 m $AgNO_3$ electrolyte (a-c). As shown, the Ag-S battery is still not reversible in these concentrated electrolytes, which should have the same failure mechanism as the 1 m electrolyte. Interestingly, the discharge capacity decreases in the order of 1 m > 2 m > 5m > 10 m, which may result from the inferior ionic conductivity and poor wetting of concentrated electrolytes.

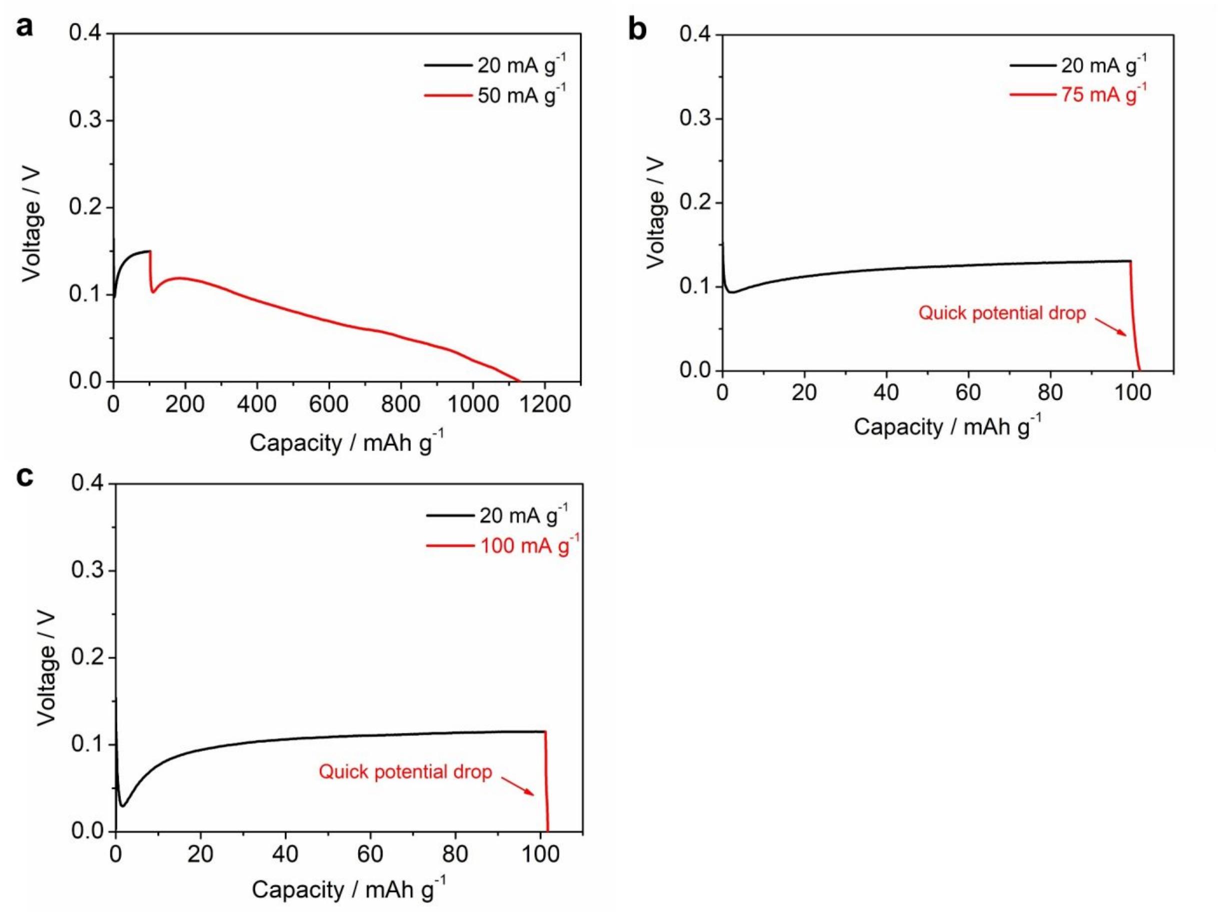


**Figure S11**. The rate performance of the Ag-S battery. (a) At 50 mA $g^{-1}$ current density; (b) At 75 mA $g^{-1}$ current density; (c) At 100 mA $g^{-1}$ current density.

**Table S1**. The comparison between representative primary and rechargeable batteries in terms of discharge time and C-rate.

| Battery types | Battery system | Applications | Discharge time | Discharge rate |
|---|---|---|---|---|
| Primary batteries | $Zn\text{-}MnO_2$ battery | Clocks | > 1 year | <0.00012 C |
| | | Wireless mouses | > 1 month | <0.0014 C |
| | | Laser pointers | > 1 month | <0.0014 C |
| | $Ag_2O\text{-}Zn$ battery | Watches | > 1 year | <0.00012 C |
| | | Calculators | > 1 year | <0.00012 C |
| Rechargeable batteries | Li-ion battery | Cellphones | 3-24 hours | 0.042-0.33 C |
| | | Laptops | 3-6 hours | 0.167-0.33 C |
| | | Electric vehicles | <10 hours | >0.1 C |

Note that some applications are used intermittently, and the discharge time is estimated as the overall usage time. As shown, most primary batteries are designed for use at low power density with an ultrasmall current rate, which is opposite to the case of rechargeable batteries.

**Table S2**. The performance comparison of different primary zinc batteries.

| Battery types | Cathode | Cathode capacity / mAh g$^{-1}$ | Cell voltage / V | Citation |
|---|---|---|---|---|
| Zn-carbon batteries | $MnO_2$ | 308 | 1.5 | [1] |
| Alkaline batteries | $MnO_2$ | 308 | 1.5 | [2] |
| Zn‖$Ag_2O$ batteries | $Ag_2O$ | 230 | 1.55 | [3] |
| Zn‖S batteries | S | 1668 | 0.70 | [4] |
| Zn‖Cu-S batteries | S | 2000 | 1.15 | [5] |
| Zn‖Ag-S batteries | S | 620 | 1.45 | This work |

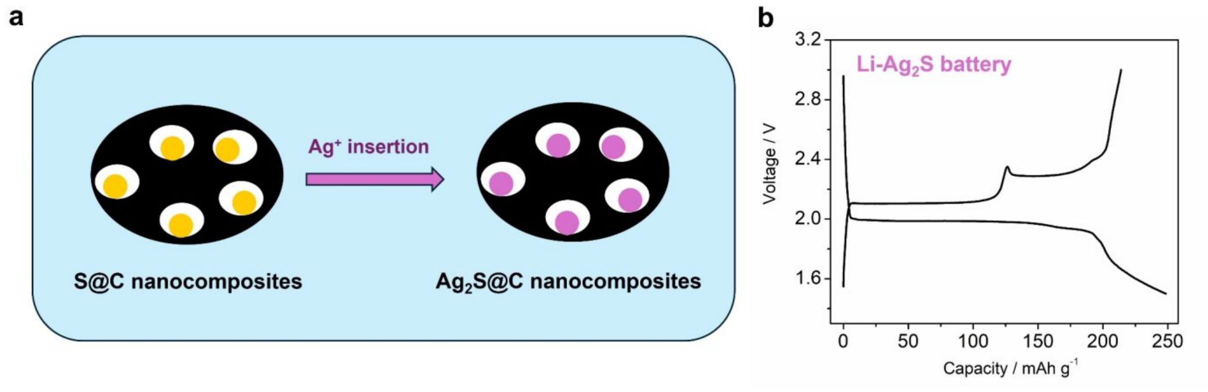


**Figure S12**. The proposed approach to synthesize $Ag_2S$ material. (a) The scheme of electrochemical preparation of $Ag_2S$@C nanocomposite in a simple aqueous medium; (b) The typical charge/discharge curve of the Li-$Ag_2S$ battery.

**Reference:**


[1] Zinc–carbon battery, *Wikipedia*, https://en.wikipedia.org/wiki/Zinc%E2%80%93carbon_battery (accessed 2023-12-03)

[2] Alkaline battery, *Wikipedia*, https://en.wikipedia.org/wiki/Alkaline_battery (accessed 2023-12-03)

[3] Fleischer, A.; Lander, J. J. *Zinc-Silver Oxide Batteries*; J. Wiley New York, **1971**

[4] Luo, L.-W.; Zhang, C.; Wu, X.; Han, C.; Xu, Y.; Ji, X.; Jiang, J.-X. A Zn–S Aqueous Primary Battery with High Energy and Flat Discharge Plateau. *Chem. Commun.* **2021**, *57* (77), 9918-9921.

[5] Wu, X.; Markir, A.; Ma, L.; Xu, Y.; Jiang, H.; Leonard, D. P.; Shin, W.; Wu, T.; Lu, J.; Ji, X. A Four-Electron Sulfur Electrode Hosting a $Cu^{2+}/Cu^{+}$ Redox Charge Carrier. *Angew. Chem.* **2019**, *131* (36), 12770-12775.
